# Differences in Toxicity Induced by Varying Degrees of Polymerization of Tristyrylphenol Ethoxylates in Male Mice

**DOI:** 10.3390/toxics13100827

**Published:** 2025-09-28

**Authors:** Chunmei Li, Fen Jin, Fengzhong Wang, Bei Fan

**Affiliations:** 1 Institute of Food Science and Technology, Chinese Academy of Agricultural Sciences, Beijing 100193, China; lichunmei@caas.cn (C.L.); wangfengzhong@sina.com (F.W.); fanbei517@163.com (B.F.); 2 State Key Laboratory for Quality and Safety of Agro-Products, Institute of Quality Standards & Testing Technology for Agro-Products, Chinese Academy of Agricultural Sciences, Beijing 100081, China

**Keywords:** tristyrylphenol ethoxylates, agricultural emulsifier #602, agricultural emulsifier #604, subacute toxicity, male mice

## Abstract

Nonylphenol ethoxylates (NPEOs) are widely utilized in pesticide formulations and industrial products but are known for their endocrine-disrupting properties. Consequently, substitutes such as tristyrylphenol ethoxylates (TSPEOs) have been introduced as inert ingredients in pesticide formulations. Here, we showed that TSPEOs exhibited subacute toxicity in male mice. For the first time, we studied the differences in subacute toxicity (28-day exposure) and the potential toxic effects of TSPEOs with varying polymerization degrees, specifically agricultural emulsifier (AE) #602 and AE #604, in male mice. We demonstrate that AE #602 can induce liver injury, as evidenced by hepatocyte swelling and vacuolar degeneration across all treated groups, along with significant hepatocellular necrosis in the high-dose group. These pathological changes were associated with alterations in oxidative stress biomarkers, including a significant decrease in malondialdehyde levels (0.57 times in the high-dose group, *p* < 0.05) and increased activities of glutathione peroxidase (up to 1.27 times, *p* < 0.05) and glutathione, suggesting a potential adaptive compensatory response. Both TSPEOs were found to cause gastric injury according to the results of organ indices and histopathological analyses. AE #604, with lower polymerization degree, caused more severe gastric injury than AE #602. Our findings indicate that NPEO substitutes should be tested for hepatotoxicity and gastrotoxicity and highlight the need for further research into the toxicity differences induced by varying degrees of polymerization of TSPEOs on human health.

## 1. Introduction

Nonylphenol ethoxylates (NPEOs) are major non-ionic surfactants extensively utilized in pesticide formulations, cleaning agents, and industrial products [1,2]. They are high-production-volume chemicals, with approximately 700,000 tons being manufactured annually globally [3]. After use and disposal, NPEOs are introduced into the environment and subsequently biodegraded into more toxic and persistent products, particularly short-chain NPEOs and nonylphenol (NP) [2,4]. Many studies have suggested that both NPEOs and their degradation product, NP, possess endocrine-disrupting properties, capable of mimicking natural hormones in various organisms [5,6]. Consequently, the commercialization and use of NPEOs and NP have been restricted or strictly monitored by the European Community [7] and the OSPAR convention [8]. In China, NPEOs and NP have been included in the draft List of Banned Pesticide Inert Ingredients (http://www.agroinfo.com.cn/other_detail_1730.html, accessed on 23 May 2025). These regulatory restrictions have driven the household, agricultural, and chemical industries to develop and implement substitutes for NPEOs.

One such NPEOs substitute is tristyrylphenol ethoxylates (TSPEOs, chemical structure depicted in Figure S1), also commonly known as agricultural emulsifier (AE) 600. A key structural feature of TSPEOs is their degree of polymerization, which varies among commercial products and may significantly influence their physicochemical properties and, consequently, their toxicological profiles. Based on the varying degrees of polymerization, the TSPEOs series includes AE #601, #602, #603, and #604, with specific product details summarized in Table S1. Owing to their effectiveness as inert ingredients, TSPEOs are extensively used in pesticide formulations, being present in 79.3% of various pesticide formulations in China [9]. Several studies have documented the widespread presence of TSPEOs in various matrices, including fruits, vegetables, oil crops, surface water, and soils [9,10,11,12,13,14,15]. Zhou et al. [9] proposed that the TSPEOs residues in fruits are associated with the application of specific pesticides during fruit growth. However, although the inert ingredients are generally considered safe, TSPEOs have recently attracted significant attention due to their potential to harm living organisms [16,17,18]. The United States Environmental Protection Agency (U.S. EPA) has reported that TSPEOs exhibit low acute oral toxicity but high subchronic toxicity, particularly affecting the kidney and thyroid in rats and the liver in dogs [16]. Subsequently, the maximum limit of TSPEOs in pesticide formulations has been set at 15% [16]. Further evidence of their toxicity was reported by Liu et al. [17], who observed that AE #602 displayed high acute toxicity to zebrafish embryos, with a median lethal concentration of 84.46 mg/L, and induced teratogenic effects such as body-lean and uninflated swim bladder in juvenile fish. Nevertheless, comparative toxicity studies across TSPEO congeners remain scarce, and how the degree of polymerization influences mammalian toxicity is still poorly understood. To date, only one study has compared the acute toxicity of various TSPEO types to *Daphnia magna*, revealing that the acute toxicity increased with increasing ethylene oxide (EO) groups, with median effective concentrations of AE #601, AE #602, and AE #603 being 36.13, 27.94, and 16.40 mg/L, respectively [18].

To address this knowledge gap, this study aimed to directly compare the subacute toxicity of two TSPEO congeners with distinct degrees of polymerization—agricultural emulsifier (AE) #602 and AE #604—and to elucidate their underlying toxicological mechanisms. AE #602 was selected for its reported higher acute toxicity in zebrafish, while AE #604 has a lower polymerization degree. We conducted a comprehensive subacute toxicity assessment in male mice, evaluating body weight, organ somatic indices, blood biochemical parameters, and histopathological analysis. The mechanisms of nephrotoxicity and hepatotoxicity related to oxidative stress were also explored using key biomarkers, including malondialdehyde (MDA), catalase (CAT), superoxide dismutase (SOD), glutathione peroxidase (GPx), and glutathione (GSH). MDA is a byproduct of lipid peroxidation and serves as an indicator of oxidative damage, whereas CAT, SOD, GPx, and GSH are critical antioxidant enzymes that mitigate oxidative damage [19,20,21,22,23]. The findings of this comparative study will enhance the understanding of how the structural attribute of polymerization degree dictates the toxicological profiles of these ubiquitous inert ingredients in pesticide formulations. Furthermore, this study aims to raise awareness regarding the potential health risks associated with NPEO substitutes and their possible adverse effects on human health, thereby contributing to the development of safer alternatives and improved regulatory guidelines.

## 2. Materials and Methods

### 2.1. Materials

Agricultural emulsifier (AE) #602 and AE #604 were obtained from Jiangsu Zhongshan Chemical Co., Ltd. (Nanjing, China). The kits of albumin (ALB), total protein (TP), aspartate transaminase (AST), alanine aminotransferase (ALT), alkaline phosphatase (ALP), total bilirubin (TBIL), blood urea nitrogen (BUN), creatinine (CR), thyroid-stimulating hormone (TSH), thyroxine (T4), triiodothyronine (T3), testosterone (T), CAT, MDA, SOD, GPx, and GSH were purchased from Nanjing Jiancheng Biological Co., Ltd. (Nanjing, China).

### 2.2. Animals

Male CD-1 mice, about 7-weeks old, were purchased from Experimental Animal Tech Co., of Weitonglihua (Beijing, China). All mice were housed under specific pathogen-free conditions at 20 ± 2 °C, with relative humidity maintained at 45–65% and exposed to artificial lighting in a 12 h light/12 h dark cycle. Throughout the experiment, all mice were provided with unrestricted access to water and standard food. The mice underwent a one-week acclimatization period to ensure their adaptation to the experimental environment before use in experiments. All animal experiments were conducted in accordance with the Health Guide for the Care and Use of Laboratory Animals of National Institutes, and animal-related protocols were approved by the Ethical Committee of Animal Use and Protection.

### 2.3. Acute Toxicity Study

Based on the U.S. EPA assessment indicating that TSPEOs are not expected to pose an acute risk (reporting a LOAEL of 1000 mg/kg/day for developmental toxicity in rats) [16], a preliminary acute-oral-toxicity limit test was performed to screen short-term severe toxicity in mice. A limit dose of 2000 mg/kg bw was selected accordingly. A total of six male mice were utilized to concurrently evaluate the two TSPEO series products (AE #602 and AE #604). Animals were randomly assigned to two treatment groups (*n* = 3 per group) and were administered either AE #602 or AE #604 via a single gavage following an overnight fast. After administration, the mice were closely observed continuously for the first hour and intermittently for the subsequent four hours. Clinical observations, including changes in fur, skin, oral mucosa, eyes, circulation, respiration, and behavior, were recorded over a two-day period to assess any signs of mortality or toxicity (note: this two-day period was shorter than the fourteen-day period required by standard protocols of GB 15193.3-2014 [24] or OECD TG 425 [25], which mandate frequent checks on day 1 and at least daily observations thereafter). Following the two-day observation period, the body weights of the mice were measured, and the animals were subsequently euthanized by 2% isoflurane inhalation and cervical dislocation.

### 2.4. Subacute Toxicity Study

The 28-day subacute toxicity assessment was performed following the national standard methods of China (GB 15193.22-2014) [26]. A total of 84 male mice were used and randomly divided into 7 groups (*n* = 12 per group). Based on the acute toxicity findings, AE #602 or AE #604 was administered to the treatment groups at doses of 33.3, 100, and 300 mg/kg/day via oral gavage for 28 days. The control group received an equivalent volume of 0.9% sodium chloride solution. Both AE #602 and AE #604 were diluted with a 0.9% sodium chloride solution to achieve the required concentrations for administration. Throughout the 28-day experimental period, all mice were monitored daily for mortality, morbidity, behavior, and any toxicity signs. Body weights were recorded on days 0, 7, 14, 21, and 29. At the end of the experiment, following a 12 h fasting period, mice were anesthetized by 2% isoflurane inhalation and subsequently underwent terminal blood collection via left ventricular puncture.

### 2.5. Organ Index Assessment

Organs including the lungs, liver, testes, brain, spleen, kidneys, heart, and stomach were carefully excised and weighed. The organ index was determined as the ratio of the organ weight to the body weight.

### 2.6. Biochemistry Analysis

Whole-blood samples were left to stand for 2 h at 37 °C and then centrifuged at 3000 rpm for 5 min to isolate serum. Serum biochemistry parameters, including TP, ALB, AST, TBIL, ALP, ALT, BUN, and CR, were detected using colorimetry. TP was determined by the Coomassie brilliant blue method (595 nm). ALB was determined by the bromocresol green method (630 nm). AST and ALT activities were measured using 2,4-dinitrophenyl hydrazine. TBIL was measured by the chemical oxidation method. ALP activity was determined by the absorbance at 520 nm. BUN was determined by the urease method. CR was quantified using the sarcosine oxidase method. T3, T4, TSH, and T were measured by an enzyme-linked immunosorbent assay. The determination methods were carried out in strict accordance with the manufacturer’s instructions provided with the corresponding kits of Nanjing Jiancheng Biological kit.

For liver and kidney tissues, homogenates were prepared by mixing the organs with saline at ratio of 1:9 (*w*/*v*). Following centrifugation at 3500 rpm for 10 min at 4 °C, the supernatants were collected for analysis. SOD was determined using the hydroxylamine method (550 nm). MDA was determined using thiobarbituric-acid-reactive substances levels (532 nm). CAT was determined by measuring the intensity of a yellow complex formed by molybdate and H_2_O_2_ at 405 nm after ammonium molybdate was added to terminate the H_2_O_2_ degradation reaction catalyzed by CAT. GPx was determined by the 5,5′dithiobis-p-nitrobenzoic acid (DTNB) method (412 nm). GSH was determined using the DTNB colorimetric method (405 nm). The Nanjing Jiancheng Biological kit’s instruction were followed while measuring all the indicators.

### 2.7. Histopathological Analysis

Collected organs were fixed in 10% buffered formalin and then subjected to dehydration, immersion, and paraffin embedding. Tissue sections (5 µm) were prepared and stained using hematoxylin and eosin for histological examination under a light microscope (IX83, Olympus Corporation, Tokyo, Japan). Organs subjected to histopathological analysis included the kidneys, liver, spleen, lungs, heart, brain, testes, and stomach.

### 2.8. Statistical Analysis

Statistical analysis was conducted using SPSS 23.0 (IBM Inc., Armonk, NY, USA). Experimental data are expressed as the mean ± standard deviation. Before conducting statistical tests, the normality of the data distribution was verified using the Shapiro–Wilk test, and the homogeneity of variances was assessed using Levene’s test. Since the data passed these assumptions (*p* > 0.05), significant differences (*p* < 0.05 and *p* < 0.01) among groups were determined using one-way ANOVA followed by Dunnett’s post hoc test. Graphical representations and additional analyses were performed with Origin 2016 software (Origin Lab, Northampton, MA, USA).

## 3. Results and Discussion

### 3.1. Acute Toxicity Study of TSPEOs

Mice administered a single oral dose of agricultural emulsifier (AE) #602 or AE #604 at 2000 mg/kg bw showed no clinical signs of toxicity or mortality within the two-day observation period. All mice appeared normal, with no alterations in behavior, breathing, skin condition, fur appearance, posture, or food and water consumption. An increase in body weight was observed in both the AE #602- and AE-#604-treated groups; the initial mean body weights were 31.2 ± 1.73 g and 32.0 ± 1.46 g, respectively, which increased to 33.1 ± 1.61 g and 33.6 ± 2.08 g by the end of the observation period. Based on the absence of adverse effects within this short-term observation window, it may be preliminarily inferred that both compounds exhibit low acute toxicity at the 2000 mg/kg dose level, which is consistent with the U.S. EPA’s assessment.

### 3.2. Subacute Toxicity Study of TSPEOs

Subacute toxicity studies are critical for evaluating the long-term safety and potential hazard of chemicals [27]. Based on the acute toxicity findings, three dosage levels (33.3, 100, and 300 mg/kg bw) were used to assess the 28-day repeated oral subacute toxicity of AE #602 and AE #604 in male mice.

#### 3.2.1. Effects of TSPEOs Exposure on Body Weight of Male Mice

**Figure 1:**
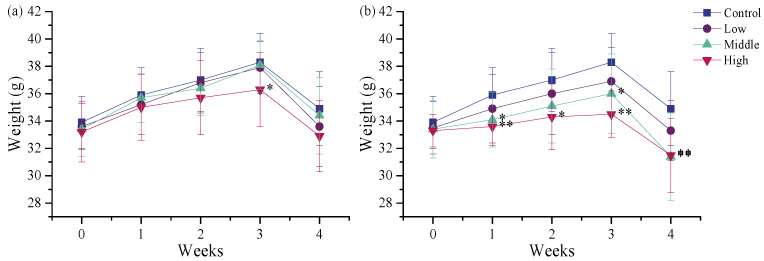
Body weights of the male mice orally administered (**a**) agricultural emulsifier (AE) #602 or (**b**) AE #604. Data are presented as the mean ± standard deviation, *n* = 12. * *p* < 0.05 and ** *p* < 0.01 compared to the control group.

During the 28-day oral exposure, no obvious clinical signs of toxicity were observed in any of the treated mice. Body weight, a key indicator of toxicity in animal studies [28], was monitored to evaluate the subacute effects of repeated exposure to different TSPEO products. Body weight in all groups increased gradually up to day 21, followed by a slight decrease at day 29, probably due to the 12 h overnight fasting (Figure 1). A dose-dependent reduction in body weight was observed. Notably, mice in the middle- and high-dose groups of AE #604 exhibited significantly (*p* < 0.05) or extremely significantly (*p* < 0.01) lower body weights compared to the control group from day 7 after administration. In contrast, for AE #602, only the high-dose group exhibited a significant reduction in body weight relative to the vehicle-treated control group on day 21 (*p* < 0.05), with no significant differences detected between the other AE #602 dose groups and the control group. The more pronounced effect of AE #604 on body weight reduction may be attributed to its lower polymerization degree. This finding aligns with previous reports on structure–activity relationships of surfactant toxicity, where shorter-chain analogues often demonstrate enhanced toxicity [29,30].

#### 3.2.2. Effects of TSPEOs Exposure on Organ Indexes of Male Mice

Organ indices provide valuable insights into microscopic alterations in specific organs [31]. In this study, organ indexes were analyzed for the lungs, liver, testes, brain, spleen, kidneys, stomach, and heart of male mice. As shown in Table 1, the lung index of mice exposed to AE #602 exhibited a dose-dependent decrease, with the middle- and high-dose groups showing significant decreases to 0.83–0.84 times that of the control group (*p* < 0.05). This reduction suggests potential lung effects, although no significant histopathological correlates were identified (as detailed in Section 3.2.5). No significant changes were observed in the indices of other organs following AE #602 exposure (*p* > 0.05). In contrast, repeated exposure to 300 mg/kg AE #604 resulted in significant increases in the liver (1.14 times, *p* < 0.05) and stomach index (1.14 times, *p* < 0.05) indices (Table 2). No notable differences were detected in the indices of the testes, lungs, brain, spleen, kidneys, and heart (*p* > 0.05). The differential organ index responses between AE #602 and AE #604 suggest structure-dependent target organ toxicity. These findings demonstrate that polymerization degree influences not only toxicity magnitude but also target organ specificity.

#### 3.2.3. Effects of TSPEOs Exposure on Serum Biochemical Parameters in Male Mice

Serum biochemical analysis is widely used to obtain information about potential organ lesions [32]. The U.S. EPA has reported that TSPEOs exposure can induce subchronic toxicity in the liver, kidney, and thyroid [16]. In this study, liver function was assessed through AST, ALB, ALP, ALT, TP, and TBIL levels; kidney function was evaluated via CR and BUN levels; thyroid function was appraised by T3, T4, and TSH levels; and testis function was measured through T levels. It can be seen from Table 3 that no statistically significant differences in serum parameters were observed between the AE #602- and AE-#604-treated groups and the control group (*p* > 0.05). However, liver function indicators such as ALT (1.29–1.54 times, *p* > 0.05) and TBIL (1.13–1.35 times, *p* > 0.05) increased in all treatment groups, while AST levels increased in the AE-#604-treated groups (1.25–1.38 times, *p* > 0.05). Elevated ALT, TBIL, and AST levels are important biomarkers of liver injury [33,34], suggesting that both AE #602 and AE #604 exert a certain influence on liver function. For kidney function indicators, CR levels increased in the high-dose groups of AE #602 and AE #604 (1.24–1.45 times, *p* > 0.05). Regarding BUN, numerical elevations were observed in the AE #602 low-dose group (1.19 times) and AE #604 high-dose group (1.22 times), but none of these changes reached statistical significance (*p* > 0.05). Elevated CR and BUN levels are indicative of impaired kidney function [35,36]; however, the lack of statistical significance suggests that the potential for kidney injury requires further verification in longer-term studies. Additionally, slight increases in thyroid function indicators T3 (1.14–1.23 times, *p* > 0.05) and T4 (1.07–1.19 times, *p* > 0.05) were observed in all treatment groups, indicating potential adverse effects on thyroid function. Although these changes did not reach statistical significance, the consistent trends across multiple parameters suggest subtle hepatorenal and thyroid effects that may become more pronounced with longer exposure durations or higher doses. The pattern of organ toxicity differs from the U.S. EPA report [16], which may be attributed to species-specific differences in the toxicity of TSPEOs.

#### 3.2.4. Effects of TSPEOs Exposure on Liver and Kidney Biochemical Parameters in Male Mice

**Figure 2:**
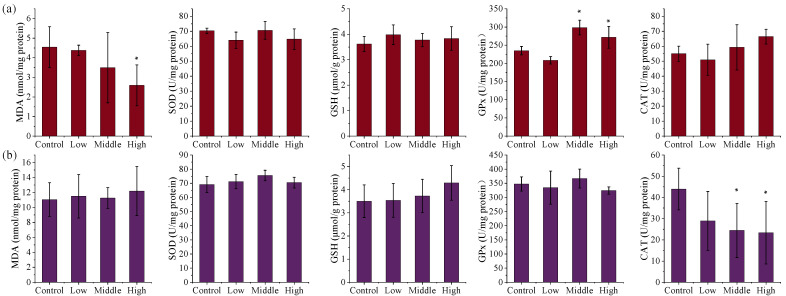
The (**a**) liver and (**b**) kidney biochemical parameters of the male mice orally administered agricultural emulsifier (AE) #602. Data are presented as the mean ± standard deviation, *n* = 12. * *p* < 0.05 compared to the control group.

Exposure to TSPEOs induced significant alterations in key oxidative stress biomarkers in the liver and kidneys of male mice (Figure 2). In the AE-#602-treated groups, SOD activity in the liver and kidneys showed no obvious changes (*p* > 0.05), while MDA levels in the liver decreased dose-dependently, with a significant reduction in the high-dose group (0.57 times, *p* < 0.05). GPx activity exhibited a significant increase in the liver (1.16–1.27 times, *p* < 0.05) in the middle- and high-dose groups, and GSH activity showed a slight increase in the liver and kidneys (*p* > 0.05). Notably, a significant, dose-dependent decrease in CAT activity was observed in the kidneys (*p* < 0.05). Collectively, these changes indicate a complex adaptive response. The suppression of CAT activity suggests an underlying oxidative challenge, which appears to be partially compensated for by the induction of the GPx/GSH system, resulting in reduced lipid peroxidation [37,38,39,40].

**Figure 3:**
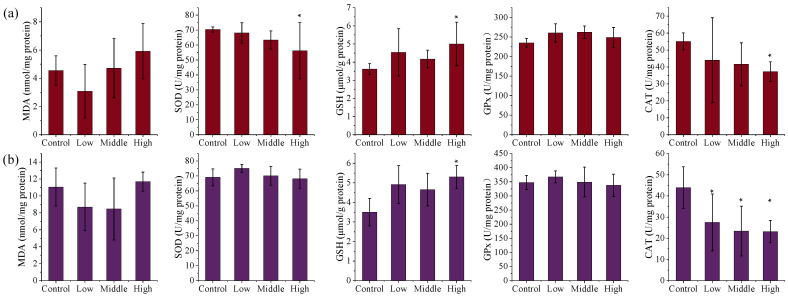
The (**a**) liver and (**b**) kidney biochemical parameters of the male mice orally administered agricultural emulsifier (AE) #604. Data are presented as the mean ± standard deviation, *n* = 12. * *p* < 0.05 compared to the control group.

In contrast, AE #604 exposure did not significantly affect the levels of MDA and GPx in the liver and kidneys (*p* > 0.05) (Figure 3). Whereas GSH levels increased dose-dependently in both tissues, with significant elevations in the high-dose group (1.38 and 1.51 times, *p* < 0.05), indicating the activation of a compensatory mechanism. The activities of CAT and SOD in the liver decreased dose-dependently, with significant reductions in the high-dose group (0.68 and 0.80 times, *p* < 0.05). Significant decreases in CAT activity in the kidneys were also observed in all AE-#604-treated groups (0.53–0.63 times, *p* < 0.05). The observed decreases in the activities of SOD and CAT thus reveal that administration of AE #604 also induces severe oxidative stress in the kidney and liver, with the liver being more sensitive than the kidneys. The distinct oxidative stress patterns induced by the two TSPEOs congeners suggest different mechanisms of toxicity. AE #602 appears to primarily induce renal oxidative stress through CAT inhibition, while AE #604 affects both liver and kidneys through suppression of SOD and CAT activities. The increased GSH levels in both treatment groups likely represent a compensatory response to counteract oxidative damage. The differential oxidative stress responses further support the structure–activity relationship between the polymerization degree and toxicological profiles of TSPEOs.

#### 3.2.5. Effects of TSPEOs Exposure on Histopathological Analysis in Male Mice

Histopathological examination is the gold standard for diagnosing disease and predicting the adverse effects of chemicals. In this study, histopathological analysis was employed to assess the toxicity of TSPEOs by examining pathological alterations in various organs. No significant alterations were detected in the kidneys, spleen, testes, brain, or heart following AE #602 or AE #604 exposure. Although a reduction in lung index was observed in the middle- and high-dose groups of AE #602, histological examination revealed only mild spontaneous background lesions (including slightly widened alveolar septa and dilated, congested capillaries) that were present at comparable levels in both treated and control mice. These findings suggest that the lung index changes lack corresponding histopathological correlates and are likely not treatment-related. Representative images of lung sections are provided in Figure S2.

**Figure 4:**
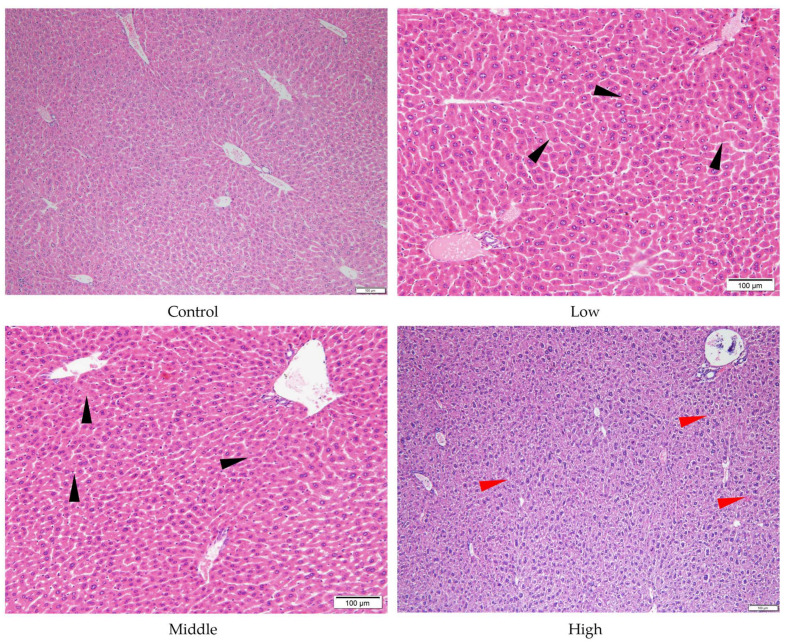
Histopathology of liver tissue of the male mice orally administered agricultural emulsifier (AE) #602. Triangles indicate representative pathological changes (black: hepatocyte swelling and granular degeneration; red: severe hepatocyte swelling and granular degeneration).

**Figure 5:**
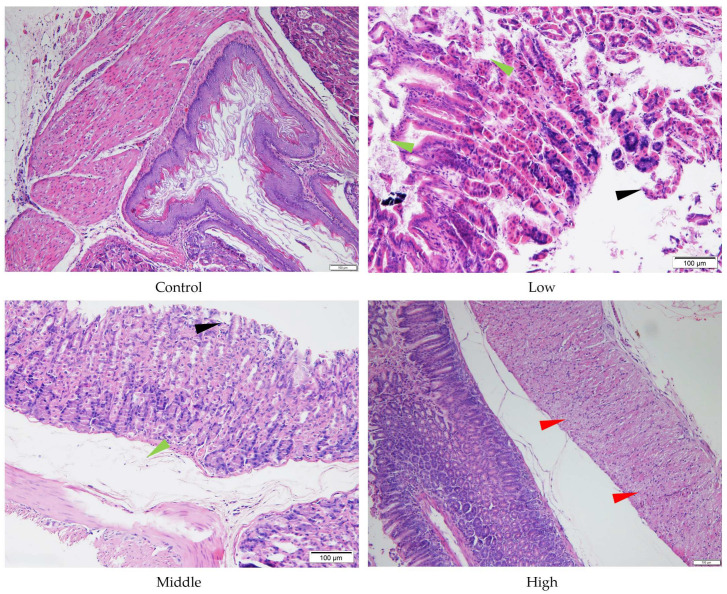
Histopathology of stomach tissue of the male mice orally administered agricultural emulsifier (AE) #602. Triangles indicate representative pathological changes (black: detachment of the glandular gastric mucosa; green: edema; red: moderate degeneration and necrosis of glandular gastric muscle cells).

**Figure 6:**
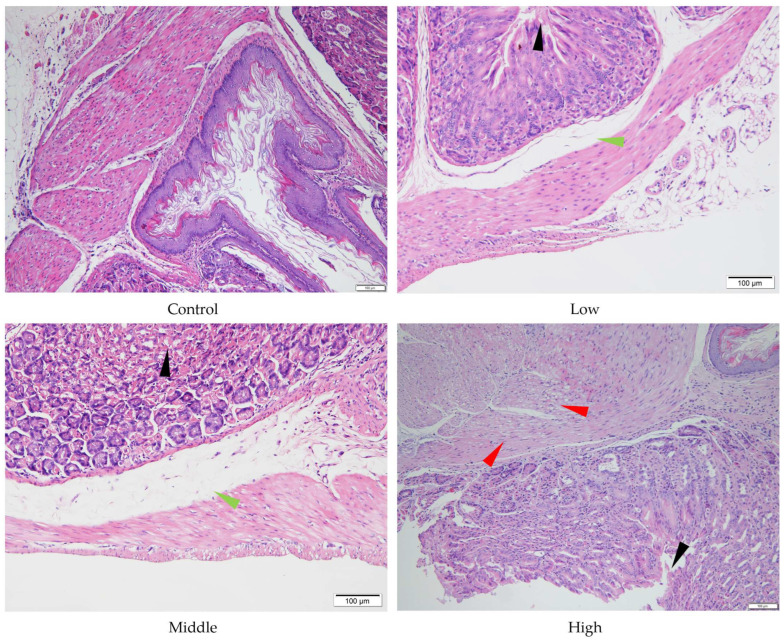
Histopathology of stomach tissue of the male mice orally administered agricultural emulsifier (AE) #604. Triangles indicate representative pathological changes (black: detachment of the glandular gastric mucosa; green: edema; red: severe muscle cell degeneration).

Despite the absence of significant liver and stomach index changes for AE #602, pathological changes were observed in these organs under a microscope. In comparison with the control group, the low- and middle-dose groups of AE #602 showed normal hepatic cord arrangement, accompanied by noticeable hepatocyte swelling and granular degeneration. In contrast, the high-dose group showed loss of hepatic cord structure, along with severe hepatocyte swelling, granular degeneration, and vacuolar degeneration. Significant hepatocellular necrosis was observed, in which the nuclei of necrotic hepatocytes had disappeared, leaving only their cellular outlines (Figure 4). These findings were consistent with the observed decreases in MDA and the increases in ALT, TBIL, and GPx values, suggesting that organ index measurements may not fully capture the functional impairment occurring at the cellular level. Gastric lesions showed a distinct pattern of toxicity. Both AE #602 and AE #604 induced slight detachment of the glandular gastric mucosa along with edema between the mucosal layer, basal layer, and muscular layer in low- and middle-dose groups. The high-dose AE #602 exhibited moderate degeneration and necrosis of glandular gastric-muscle cells (Figure 5). For AE #604, the significant increase in stomach indexes in the high-dose group was corroborated by substantial histopathological findings, including severe muscle-cell degeneration, mucosal epithelial cell detachment, and gastric mucosal ulceration with inflammation (Figure 6), indicating active inflammation and tissue destruction that directly explains the increased stomach index. These findings indicate that AE #602 administration can lead to liver damage in mice, while AE #604, with its lower polymerization degree, results in more severe gastric injury compared to AE #602. The incidence and severity of histopathological lesions are presented in Table S2. These organ-specific toxicity profiles reveal important structure–activity relationships that help explain the differential organ index responses.

These results contrast with the U.S. EPA’s report [16], which identified high subchronic toxicity of TSPEOs in the thyroid and kidneys in rats and the liver in dogs. These differences may be attributed to species differences. Furthermore, our findings differ from prior research by Li et al. [18], who reported that the acute toxicity of TSPEOs to *Daphnia magna* increased with increasing EO groups, highlighting the importance of exposure duration and model organisms in toxicity assessment. Notably, similar to the pattern observed in NPEOs, where short-chain NPEOs are more toxic than long-chain NPEOs [2,4,41,42], the lower polymerization degree of AE #604 was associated with more pronounced gastric toxicity in this study. The discrepancy between organ index changes and histopathological findings—particularly the lack of correlation between lung weight reduction and histological changes in AE #602 groups—emphasizes the critical importance of integrating gross pathology, organ weights, and microscopic examination in toxicological assessment.

Overall, these findings demonstrate that polymerization degree significantly influences both toxicity magnitude and target organ specificity in male mice, with AE #604 exhibiting more pronounced effects on organs showing index changes (stomach), while AE #602 causes significant histological damage in organs without corresponding index changes (liver), highlighting the complex relationship between organ weight alterations and actual tissue damage.

### 3.3. Limitations of the Study

A limitation of this study is the exclusive use of male mice, which prevents the assessment of sex-specific toxicity of AE #602 and AE #604. Given that sex differences in metabolism and tissue responsiveness may lead to distinct toxicological outcomes, future studies should include both male and female animals to fully characterize the safety profiles of AE #602 and AE #604. Additionally, while this study identified oxidative stress as a contributing mechanism to TSPEO-induced hepatotoxicity, the underlying molecular initiating events remain unclear. Specifically, the potential roles of peroxisome-proliferator-activated receptor activation, peroxisome proliferation, or induction of specific cytochrome P450 enzymes were not investigated. Further studies incorporating targeted assays—such as measuring acyl-coenzyme A oxidase activity for peroxisomal proliferation or specific CYP450 activities (e.g., 7-ethoxyresorufin-O-deethylase, 7-benzyloxyresorufin-O-debenzylase, and/or 7-pentoxyresorufin-O-depentylase activities)—are necessary to fully elucidate the mode of action.

## 4. Conclusions

This study represents the first comparative analysis of the toxicity of TSPEOs with varying polymerization degrees in male mice. Subacute-toxicity tests revealed that agricultural emulsifier (AE) #602 could induce liver injury through oxidative stress. Both TSPEOs were found to cause gastric injury, with AE #604, characterized by a lower polymerization degree, resulting in more severe gastric damage compared to AE #602. These findings hint at the potential health risks associated with TSPEOs, raising concerns about their safety in pesticide formulations applied to agricultural products. Our results suggest that TSPEOs and their potential negative impacts on human health deserve further research, particularly regarding the toxicity differences arising from varying polymerization degrees. Furthermore, this work raises concerns about the safety of NPEO alternatives and the present toxicological management of substitutes for hazardous inert ingredients used in pesticide formulations.

## Figures and Tables

**Table 1:** Organ weights and indices of the male mice orally administered agricultural emulsifier (AE) #602.

Organ	Parameters	Control	Low	Middle	High
Lung	Weight (g)	0.280 ± 0.074	0.239 ± 0.036 *	0.231 ± 0.028 *	0.218 ± 0.024 **
	Index (%)	0.797 ± 0.176	0.717 ± 0.136	0.673 ± 0.083 *	0.663 ± 0.058 *
Liver	Weight (g)	1.608 ± 0.205	1.525 ± 0.304	1.589 ± 0.282	1.441 ± 0.276
	Index (%)	4.605 ± 0.463	4.501 ± 0.539	4.612 ± 0.606	4.360 ± 0.624
Testis	Weight (g)	0.232 ± 0.033	0.276 ± 0.029 **	0.248 ± 0.037	0.229 ± 0.032
	Index (%)	0.666 ± 0.106	0.828 ± 0.116 **	0.722 ± 0.092	0.697 ± 0.090
Brain	Weight (g)	0.473 ± 0.057	0.457 ± 0.060	0.477 ± 0.040	0.446 ± 0.063
	Index (%)	1.355 ± 0.139	1.368 ± 0.222	1.397 ± 0.165	1.361 ± 0.212
Spleen	Weight (g)	0.108 ± 0.071	0.090 ± 0.015	0.104 ± 0.028	0.103 ± 0.030
	Index (%)	0.318 ± 0.242	0.268 ± 0.036	0.302 ± 0.088	0.314 ± 0.093
Kidney	Weight (g)	0.568 ± 0.090	0.538 ± 0.084	0.572 ± 0.104	0.501 ± 0.063 *
	Index (%)	1.626 ± 0.221	1.598 ± 0.195	1.666 ± 0.287	1.524 ± 0.168
Stomach	Weight (g)	0.257 ± 0.032	0.239 ± 0.046	0.248 ± 0.036	0.260 ± 0.041
	Index (%)	0.737 ± 0.069	0.711 ± 0.126	0.723 ± 0.091	0.789 ± 0.094
Heart	Weight (g)	0.179 ± 0.029	0.172 ± 0.027	0.184 ± 0.029	0.165 ± 0.015
	Index (%)	0.514 ± 0.084	0.511 ± 0.074	0.540 ± 0.107	0.503 ± 0.037

Data are presented as the mean ± standard deviation, *n* = 12. * *p* < 0.05 and ** *p* < 0.01 compared to the control group.

**Table 2:** Organ weights and indices of the male mice orally administered agricultural emulsifier (AE) #604.

Organ	Parameters	Control	Low	Middle	High
Lung	Weight (g)	0.280 ± 0.074	0.237 ± 0.028 *	0.257 ± 0.029	0.238 ± 0.036 *
	Index (%)	0.797 ± 0.176	0.713 ± 0.072	0.824 ± 0.094	0.758 ± 0.142
Liver	Weight (g)	1.608 ± 0.205	1.580 ± 0.283	1.511 ± 0.337	1.671 ± 0.351 *
	Index (%)	4.605 ± 0.463	4.739 ± 0.717	4.780 ± 0.645	5.244 ± 0.670 *
Testis	Weight (g)	0.232 ± 0.033	0.241 ± 0.052	0.235 ± 0.037	0.225 ± 0.038
	Index (%)	0.666 ± 0.106	0.725 ± 0.149	0.753 ± 0.112	0.712 ± 0.109
Brain	Weight (g)	0.473 ± 0.057	0.449 ± 0.030	0.460 ± 0.036	0.463 ± 0.049
	Index (%)	1.355 ± 0.139	1.354 ± 0.120	1.477 ± 0.132	1.483 ± 0.243
Spleen	Weight (g)	0.108 ± 0.071	0.097 ± 0.030	0.099 ± 0.028	0.089 ± 0.024
	Index (%)	0.318 ± 0.242	0.291 ± 0.077	0.314 ± 0.078	0.279 ± 0.062
Kidney	Weight (g)	0.568 ± 0.090	0.512 ± 0.067	0.488 ± 0.060 **	0.505 ± 0.053 *
	Index (%)	1.626 ± 0.221	1.536 ± 0.154	1.558 ± 0.147	1.606 ± 0.195
Stomach	Weight (g)	0.257 ± 0.0320	0.250 ± 0.034	0.252 ± 0.049	0.265 ± 0.045
	Index (%)	0.737 ± 0.069	0.754 ± 0.096	0.804 ± 0.121	0.840 ± 0.123 *
Heart	Weight (g)	0.179 ± 0.029	0.163 ± 0.022	0.173 ± 0.040	0.171 ± 0.042
	Index (%)	0.514 ± 0.084	0.490 ± 0.055	0.554 ± 0.125	0.540 ± 0.122

Data are presented as the mean ± standard deviation, *n* = 12. * *p* < 0.05 and ** *p* < 0.01 compared to the control group.

**Table 3:** Serum biochemical parameters of the male mice orally administered agricultural emulsifier (AE) #602 and AE #604.

Parameters	Control	AE #602			AE #604		
		Low	Middle	High	Low	Middle	High
TP (g/L)	45.0 ± 2.3	42.9 ± 4.1	44.5 ± 3.5	44.4 ± 1.5	40.7 ± 2.2	42.6 ± 2.1	47.1 ± 5.1
ALB (g/L)	26.4 ± 2.8	25.7 ± 3.1	27.2 ± 2.0	28.8 ± 1.1	24.7 ± 2.8	28.2 ± 0.81	29.6 ± 3.6
ALP (U/L)	60.9 ± 16	53.9 ± 4.4	57.1 ± 12.0	52.4 ± 7.8	57.1 ± 16	64.5 ± 15	69.0 ± 19.0
ALT (U/L)	6.41 ± 1.7	9.62 ± 4.0	8.27 ± 3.5	9.33 ± 1.2	9.9 ± 3.4	9.12 ± 3.9	9.07 ± 2.9
AST (U/L)	8.39 ± 2.2	9.49 ± 2.5	9.65 ± 3.9	6.93 ± 1.6	10.8 ± 5.1	10.5 ± 3.5	11.6 ± 2.9
T-BIL (μmol/L)	3.62 ± 1.0	4.24 ± 1.6	4.13 ± 1.3	4.88 ± 2.1	4.24 ± 1.1	4.22 ± 0.7	4.1 ± 2.9
Cr (μmol/L)	21.5 ± 13.0	24.7 ± 7.5	23.7 ± 12.0	26.7 ± 11.0	19.5 ± 3.5	21.1 ± 9.9	31.1 ± 8.3
BUN (mmol/L)	2.23 ± 0.28	2.66 ± 0.28	2.35 ± 0.94	2.18 ± 0.4	2.59 ± 0.61	2.15 ± 0.38	2.72 ± 0.36
T3 (ng/mL)	29.1 ± 5.7	34.8 ± 6.5	34.6 ± 9.0	35.4 ± 3.6	35.9 ± 5.4	33.3 ± 3.9	33.7 ± 6.3
T4 (ng/mL)	117 ± 20.0	127 ± 21	134 ± 15.0	135 ± 8.5	127 ± 31	125 ± 12.0	139 ± 19.0
TSH (ng/mL)	22.8 ± 2.0	21.7 ± 1.5	21.9 ± 2.1	22.6 ± 2.8	21.8 ± 3.9	21.7 ± 2.1	20.2 ± 1.6
T (ng/mL)	3.15 ± 0.5	2.96 ± 0.34	2.87 ± 0.5	3.16 ± 0.54	2.96 ± 0.7	2.95 ± 0.11	2.58 ± 0.21

Data are presented as the mean ± standard deviation, *n* = 12.

## Data Availability

Data are contained within the article.

## Supplementary Materials

The following supporting information can be downloaded at: https://www.mdpi.com/article/10.3390/toxics13100827/s1, Figure S1: Molecular structure of TSPEOn (where *n* indicates the number of ethoxylate units); Figure S2: Histopathology of lung tissue of the male mice orally administered agricultural emulsifier (AE) #602; Table S1: Basic information of the agricultural emulsifier 600 series products; Table S2: Incidence and severity of histopathological lesions in the liver and stomach of male mice treated with agricultural emulsifier (AE) #602 and AE #604.
