# Supplementary material for: Differences in Toxicity Induced by Varying Degrees of Polymerization of Tristyrylphenol Ethoxylates in Male Mice
Source: Toxics. 2025 Sep 28;13(10):827. doi: 10.3390/toxics13100827 (PMC12567951; doi:10.3390/toxics13100827)
Supplement: Supplementary file 1 [file toxics-13-00827-s001.zip › toxics-3876643-supplementary.pdf]

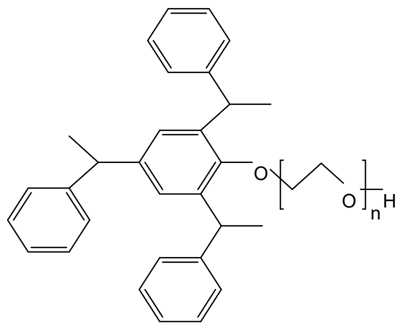

Figure S1. Chemical structure of TSPEO*n* (where *n* is the number of ethoxylate units).

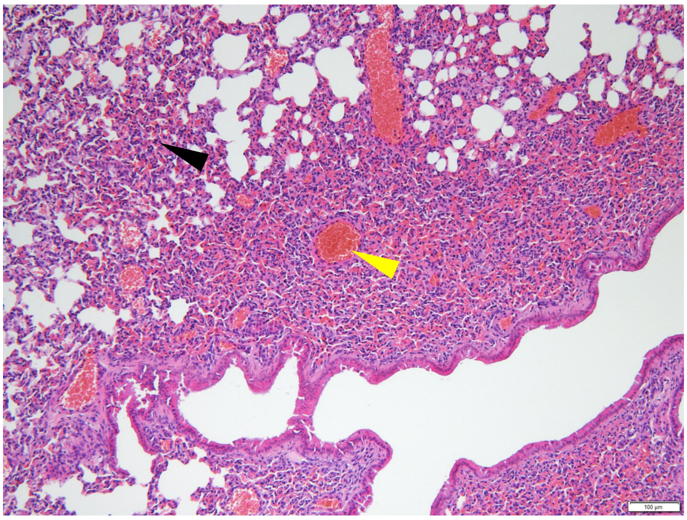

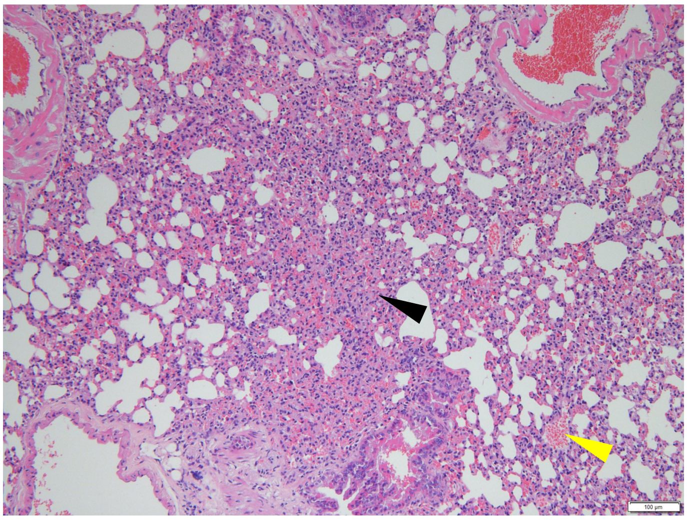

Control Low

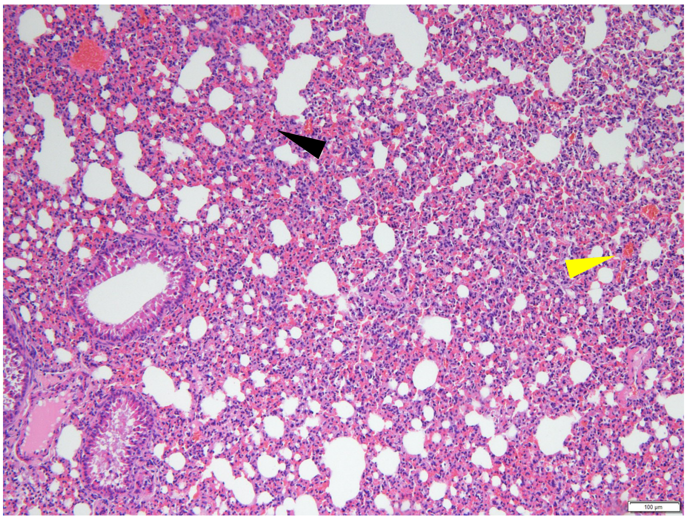

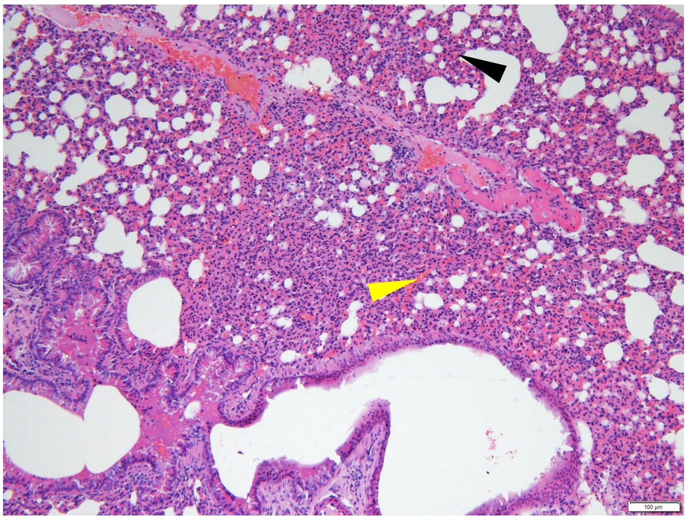

Middle High

Figure S2. Histopathology of lung tissue of the male mice orally administered agricultural emulsifier (AE) #602. Triangles indicated representative pathological changes (black: widened alveolar septa; yellow: dilated, congested capillaries)

Table S1. Basic information of the agricultural emulsifier (AE) 600 series products.

Products	Cloud point (°C)	Average of ethoxylate units
AE #601	60-65	16
AE #602	80-85	20
AE #603	90-95	21
AE #604	54-60	10

Table S2. Incidence and severity of histopathological lesions in the liver and stomach of male mice treated with agricultural emulsifier (AE) #602 and AE #604.

Findings	Grade	Groups						
		Control	AE #602			AE #604		
			Low	Middle	High	Low	Middle	High
Liver								
Hepatocyte swelling	−	12	0	0	0	11	10	11
	+	0	4	0	0	1	2	1
	++	0	8	8	2	0	0	0
	+++	0	0	4	10	0	0	0
Hepatocyte degeneration	−	12	0	0	0	12	12	11
	+	0	2	0	0	0	0	1
	++	0	10	9	4	0	0	0
	+++	0	0	3	8	0	0	0
Hepatocellular necrosis	−	12	12	11	0	12	12	12
	+	0	0	1	1	0	0	0
	++	0	0	0	9	0	0	0
	+++	0	0	0	3	0	0	0
Stomach								
Detachment of the glandular gastric mucosa	−	12	9	7	0	8	8	0
	+	0	3	5	0	4	4	0
	++	0	0	0	0	0	0	0
	+++	0	0	0	12	0	0	12
Edema	−	12	0	0	0	1	0	0
	+	0	12	12	4	11	12	3
	++	0	0	0	8	0	0	9
	+++	0	0	0	0	0	0	0
Degeneration of glandular gastric muscle cells	−	12	12	9	0	12	10	0
	+	0	0	2	0	0	2	0
	++	0	0	1	3	0	0	2
	+++	0	0	0	9	0	0	10
Necrosis of glandular gastric muscle cells	−	12	12	11	0	12	11	0
	+	0	0	1	0	0	1	0
	++	0	0	0	2	0	0	1
	+++	0	0	0	10	0	0	11

Grades are as follows: –, normal; +, slight; ++, moderate; and +++, severe change
